# Case Report: Paradoxical acrodermatitis of Hallopeau-like eruption following anti-IL-17 therapy

**DOI:** 10.12688/f1000research.18493.1

**Published:** 2019-03-26

**Authors:** Giulia Tadiotto Cicogna, Francesco Messina, Linda Nalotto, Serena Szekely, Mauro Alaibac

**Affiliations:** 1 Unit of Dermatology, Department of Medicine, University of Padua, Padova, 35121, Italy; 2 Unit of Reumathology, Department of Medicine, University of Padua, Padova, 35121, Italy

**Keywords:** IL-17, psoriasis, Acrodermatitis continua of Hallopeau, paradoxical reaction

## Abstract

Psoriasis is a chronic immune-mediated inflammatory disease. Up to 40% of patients with psoriasis may develop psoriatic arthritis.  Currently, interleukin (IL)-17/IL-23 pathways are identified as key factors in the immunopathogenesis of both conditions. Here we describe the case of a patient who developed psoriasiform skin lesions 10 months after the initiation of anti-IL17 therapy for psoriatic arthritis. The underlying disease had responded well to the therapy, but the patient developed a striking pustular eruption at the fingers with nail involvement, onycholysis, yellow discoloration, and subungual keratosis. Clinical and histological findings were consistent with an acrodermatitis continua of Hallopeau-like eruption. Skin lesions subsided after discontinuation of the responsible anti-IL17 agent. The interpretation of this paradoxical side effect of biological therapies remains unclear but may relate to an unbalanced inflammatory cytokine response induced by the inhibition of TNF activity. It is likely that patients, who are genetically prone, may respond exaggeratedly to a cytokine imbalance. The identification of this kind of patient, in the future, could be useful in order to choose the correct therapy.

## Introduction

Psoriasis is an immune-mediated inflammatory disease characterized by a chronic course and a systemic involvement. Numerous cytokines are involved in the pathogenesis of psoriasis, but the interleukin (IL)-23/17 axis has been identified as one of the key pathways ^1^. Up to 40% of patients with psoriasis may develop psoriatic arthritis (PsA). Both conditions share common pathogenic mechanisms ^2^. Among the therapies that can be used for both skin and joint manifestations, anti-TNF agents and new biologics targeting IL-17 have shown impressive efficacy ^3^. We report the case of a patient who presented with an acrodermatitis continua of Hallopeau (ACH)-like paradoxical reaction to anti-IL17 therapy for PsA.

## Case report

**Figure 1.**
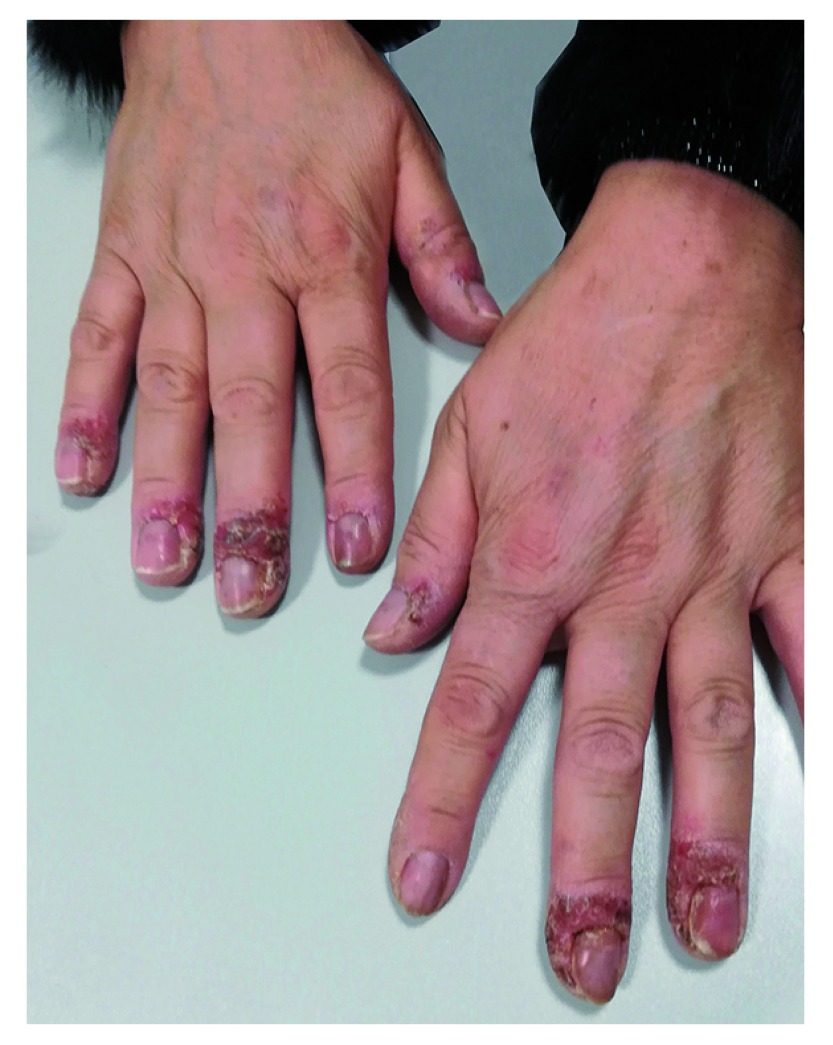
Eruption of pustules with scaling, tender swelling over fingers of both hands in a patient with psoriatic arthritis.

A 52-year-old-woman affected by PsA presented to our Dermatology Unit complaining of a painful eruption of pustules with scaling and tender swelling on the fingers of both hands ( Figure 1), which had begun one month before. Her medical history revealed concurrent PsA in complete remission after 10 months of the, the anti-IL-17 drug, secukinumab, at the dosage of 150mg every 4 weeks. The patient did not suffer from any other relevant disease and there was no family history of psoriasis.

A skin biopsy was taken, and the subsequent histopathological examination showed a stratified squamous epithelium with parakeratosis, hyperkeratosis and irregular elongation of the rete ridges of the epidermis with some lymphocytes and subcorneal collections of neutrophils forming spongiform pustules of Kogoj. This result, together with clinical features and negative results of multiple cultures confirmed our suspect of an ACH-like eruption ^4^.

It was then decided to stop anti-IL-17 therapy. A subsequent treatment plan comprised topical clobetasol propionate, once daily, and acitretin 10mg daily.

At the follow-up visit 2 months later, the lesions had visibly regressed and the patient referred a 70% reduction in symptoms measured with Dermatology Life Quality Index questionnaire. The interruption of secukinumab notwithstanding, PsA showed no recrudescence.

## Discussion

The natural history of psoriasis has been modified in the last years by new biologic agents that have allowed specific targeting of key cytokines such as TNF alpha, IL-12, IL-23 and IL-17.

TNF alpha inhibitors are the oldest, and therefore most studied, biologic drugs that have been introduced in the therapy of psoriasis. By inhibiting the whole pathway, TNF alpha blockers determine a stronger alteration of cytokine network ^5^. This has been associated not only with impressive efficacy, but also remarkable side effects, such as infections, autoimmune diseases, lymphomas and cutaneous adverse events, mainly represented by paradoxical psoriasis ^6^.

In the last years, new biologic therapies targeting cytokines situated downstream to TNF alpha, such as ustekinumab, secukinumab and, more recently, ixekizumab have shown even higher efficacy due to their selectivity in blocking cytokines specific to the pathogenic pathways. Also for this drug, although uncommon, important adverse reactions such as opportunistic infections, can be observed ^7^.

In the literature, one case of paradoxical psoriasic reaction has recently been described in association to secukinumab ^8^. In our case report we have observed an unexpected case of paradoxical ACH-like eruption, which to the best of our knowledge, has not been described in literature as an adverse event of secukinumab.

It is possible that secukinumab, by blocking IL-17, has induced a rearrangement of the cytokine pattern in this patient, determining a paradoxical increase of other pathogenic molecules, such as TNF alpha. This hypothesis has already been suggested for paradoxical reactions to ustekinumab, which exerts a blockade of IL12 and IL23 ^9^. On the other hand, is also possible that the inhibition of IL17 induced a negative feedback in the IL23-IL17 axis, thus determining an increase of IL23 which, in turn, stimulated Th17 cells to produce other cytokines, such as IL-22, which also exerts proliferation and activation of keratinocytes. Activated keratinocytes could induce the chemotaxis of neutrophils (IL-8 etc), causing the clinical presentation that we have observed in our patient ^10^. This is the first case report describing ACH-like lesions induced by secukinumab; therefore few studies are available in the literature elucidating the pathogenesis of this paradoxical reaction. Hence, our experience needs to be reinforced by further investigations.

It is likely that our patient was genetically prone to respond exaggeratedly to a cytokine imbalance. The distinction of such patients could be useful in the future, in order to predict this type of adverse reaction and, therefore, suggesting them an alternative to biologic therapies.

## Consent

Written informed consent for publication of their clinical details and/or clinical images was obtained from the patient.

## Data availability

All data underlying the results are available as part of the article and no additional source data are required.
