# Supplementary material for: Case Report: Paradoxical acrodermatitis of Hallopeau-like eruption following anti-IL-17 therapy
Source: F1000Res. 2019 Mar 26;8:336. [Version 1] doi: 10.12688/f1000research.18493.1 (PMC6685451; doi:10.12688/f1000research.18493.1)
Supplement: Peer review history

# Peer review: Reviewer response for version 1

**DOI:** 10.5256/f1000research.20235.r51595

**Published:** 2019-08-05

**Authors:** Melinda J. Gooderham

**Affiliations:** 1 SKiN Centre for Dermatology, Peterborough, ON, Canada

An interesting case of a 'paradoxical' reaction to a biologic agent, secukinumab, which is used to treat psoriatic disease. In this case, acrodermatitis of Hallopeau developed in this patient being treated successfully with secukinumab for psoriatic arthritis. It is important to report these rare events as we build our knowledge of real-world experience of the use of biologic agents in psoriatic disease.

 However, this is not the first report of this type of reaction to secukinumab in the literature. Sladden *et al*. ^1^ reported a similar reaction of fingertip and nail psoriatic disease (ACH-like) after treatment of plaque psoriasis with secukinumab. The similarity in the presentation of these cases is quite interesting and should be noted. Similar to the story of 'paradoxical' psoriasis/psoriasiform eruption with TNF antagonists, as we use more IL-17 inhibitors in the real world, we can better categorize these unexpected eruptions and further research can help to determine the underlying pathophysiological relationship.

I confirm that I have read this submission and believe that I have an appropriate level of expertise to confirm that it is of an acceptable scientific standard.

## References

[1] : Secukinumab-Induced Psoriasiform Eruption. *JAMA Dermatology*.2017;153(11) : 10.1001/jamadermatol.2017.2826 10.1001/jamadermatol.2017.2826 28768308

# Peer review: Reviewer response for version 1

**DOI:** 10.5256/f1000research.20235.r48170

**Published:** 2019-05-15

**Authors:** Archana Gopalakrishnan

**Affiliations:** 1 University of Maryland, Baltimore, Baltimore, MD, USA; 2 Rutgers University - The State University of New Jersey, New Brunswick, NJ, USA

Paradoxical effects of anti-cytokine therapy is a very important area that the authors have highlighted in this case study. As has been well documented, the use of therapies, such as anti-TNF, although groundbreaking does raise a multitude of issues - including higher risk to infections - specifically tuberculosis. The authors have documented well that the patient who was on anti-IL-17 therapy did not suffer from any infections, an important advantage of using secukinumab.

 Some of the points that could have been elaborated:

1. Would decreasing the dosage of the drug (from 150mg every 4 weeks) been able to reduce the ACH like eruption along with treating PsA? It would be useful if the authors could reference successful cases, if any documented, where the drug has been functional at lower doses.
2. A cytokine imbalance leading to an increase in TNF during secukinumab treatment has been suggested - is there corroborating evidence that implies this? If blood samples from the patient has been saved, can systemic levels of TNF be assayed for?
3. The authors raised an excellent point about underlying genetics contributing to the potential cytokine imbalance. This might be a very crucial finding and if so, could the authors highlight the nature of the polymorphisms to look for in future cases?

I confirm that I have read this submission and believe that I have an appropriate level of expertise to confirm that it is of an acceptable scientific standard.
